# PDBFlex: exploring flexibility in protein structures

**DOI:** 10.1093/nar/gkv1316

**Published:** 2015-11-28

**Authors:** Thomas Hrabe, Zhanwen Li, Mayya Sedova, Piotr Rotkiewicz, Lukasz Jaroszewski, Adam Godzik

**Affiliations:** Bioinformatics and Systems Biology Program, Sanford Burnham Prebys Medical Discovery Institute, 10901 North Torrey Pines Road, La Jolla, CA 92037, USA

## Abstract

The PDBFlex database, available freely and with no login requirements at http://pdbflex.org, provides information on flexibility of protein structures as revealed by the analysis of variations between depositions of different structural models of the same protein in the Protein Data Bank (PDB). PDBFlex collects information on all instances of such depositions, identifying them by a 95% sequence identity threshold, performs analysis of their structural differences and clusters them according to their structural similarities for easy analysis. The PDBFlex contains tools and viewers enabling in-depth examination of structural variability including: 2D-scaling visualization of RMSD distances between structures of the same protein, graphs of average local RMSD in the aligned structures of protein chains, graphical presentation of differences in secondary structure and observed structural disorder (unresolved residues), difference distance maps between all sets of coordinates and 3D views of individual structures and simulated transitions between different conformations, the latter displayed using JSMol visualization software.

## INTRODUCTION

The PDBFlex database was developed to facilitate analysis of intrinsic flexibility of protein structures as revealed by structural variations between different occurrences of the same protein chain in the Protein Data Bank (PDB – http://www.rcsb.org, version from 23 April 2015) (1). It is also a resource for protein modelers, allowing them to easily identify regions and types of flexibility in protein families. Our group has previously analyzed protein structural variability based on differences between experimentally characterized structures of the same protein in the context of identifying regions that undergo order-disorder transition (2), followed by a large scale analysis of such flexibilities in all of PDB (3). PDBFlex provides an easy access to the information used in these analyses, expanded by additional visualization and analysis tools.

The PDB database contains over 100 000 sets of coordinates (4), but only slightly over 10 000 unique protein chains. A simple comparison of these two numbers indicates that there is a lot of redundancy in PDB depositions, with most proteins being solved multiple times in independent experiments. Interestingly, in many cases there are substantial differences between structures of the same protein solved under different conditions. This variability cannot be explained by experimental errors and the observed differences reflect structural differences between functional states such as apo and holo forms (5), changes related to physicochemical conditions during crystallization or crystal packing in different crystal forms or simply reflecting the breadth of the conformational ensemble of a single protein structure (6). Understanding and cataloging such structural changes may help us understand the mechanism of enzymatic catalysis or features influencing it (7), mechanisms of allosteric regulations (8) and many other phenomena. From a practical point of view, the analysis of structural variability is helpful in assessing local reliability of protein models and in selecting models representing the right structural variant for the functional state we're interested in.

The term structural flexibility may refer to structural disorder (i.e. regions that are usually not directly observable in X-ray structures), to conformational transition between two or more structures or to ‘evolutionary flexibility’ in structures of homologous but not identical proteins. Here, we use this term to denote actual differences between distinct structures of the same protein (it is close but not necessarily identical to the second meaning mentioned above).

The task of extracting all instances of the same protein chain from PDB, superimposing them, analyzing their structural differences and grouping them according to their structural similarities and ligands is tedious, time-consuming and error prone if done manually. Especially for protein families with large representation in PDB, such as protein kinases, the task of collecting open, closed and intermediate conformations requires a lot of manual comparisons and literature analysis.

The PDBflex resource aims at addressing this problem. Structures of protein chains with identical sequences (including different protein chains from the same deposition) were collected, superimposed and global and local structural differences between them are summarized numerically and graphically using tools and viewers enabling in-depth examination. PDBFlex is focused on structural changes in identical protein chains rather than changes linked to sequence divergence but we used a 95% identity cutoff to allow for few natural or engineered substitutions in compared chains. With some notable exceptions (substitutions involving proline and glycine residues located in loops) such substitutions should not cause big conformational changes by themselves. The graphical presentations of the PDBFlex dataset include: 2D-scaling visualization of RMSD distances between structures of the same protein, graphs of average local RMSD in the aligned structures of a protein chain, visualization of the difference distance maps, graphical presentation of differences in secondary structure and observed structural disorder (unresolved residues), and JSmol-based 3D views of individual structures. Users can browse and filter the PDBFlex database and search the clusters by entering a PDB id or by sequence similarity (via BLAST search started with a sequence provided by a user).

Very few servers with similar functionality can be found in literature, such as the now defunct PCDB database of the same protein in multiple conformations (9) and its new incarnation, the CoDNaS database (10). However, neither PCDB nor CoDNaS provide any visualization nor any ways of analyzing the flexibility, providing only RMSD between different sets of coordinates. Information about some examples of structural rearrangements in proteins is available from the MolMovDB server and database (11), but PDBFlex provides automated analyses for all PDB coordinates. The collection of methods presented in Struster (12) aims to detect conformational changes in similar proteins from structural information, but it is not based on clustering of entire PDB but, instead, relies on SCOP structural classification so users are restricted to domains annotated in SCOP. The unique features of PDBFlex as compared to those databases are: the distinction made between local and global structural flexibility and inclusion of all X-ray structures from PDB.

The PDBflex database is publicly available at http://pdbflex.org for all users without a login requirement. PDBFlex was developed and tested with the major browsers (Chrome, Firefox, Safari) and operating systems (Win8, OSX, Debian).

## USING PDBFlex

### Finding the information about flexibility of a specific protein

**Figure 1.**
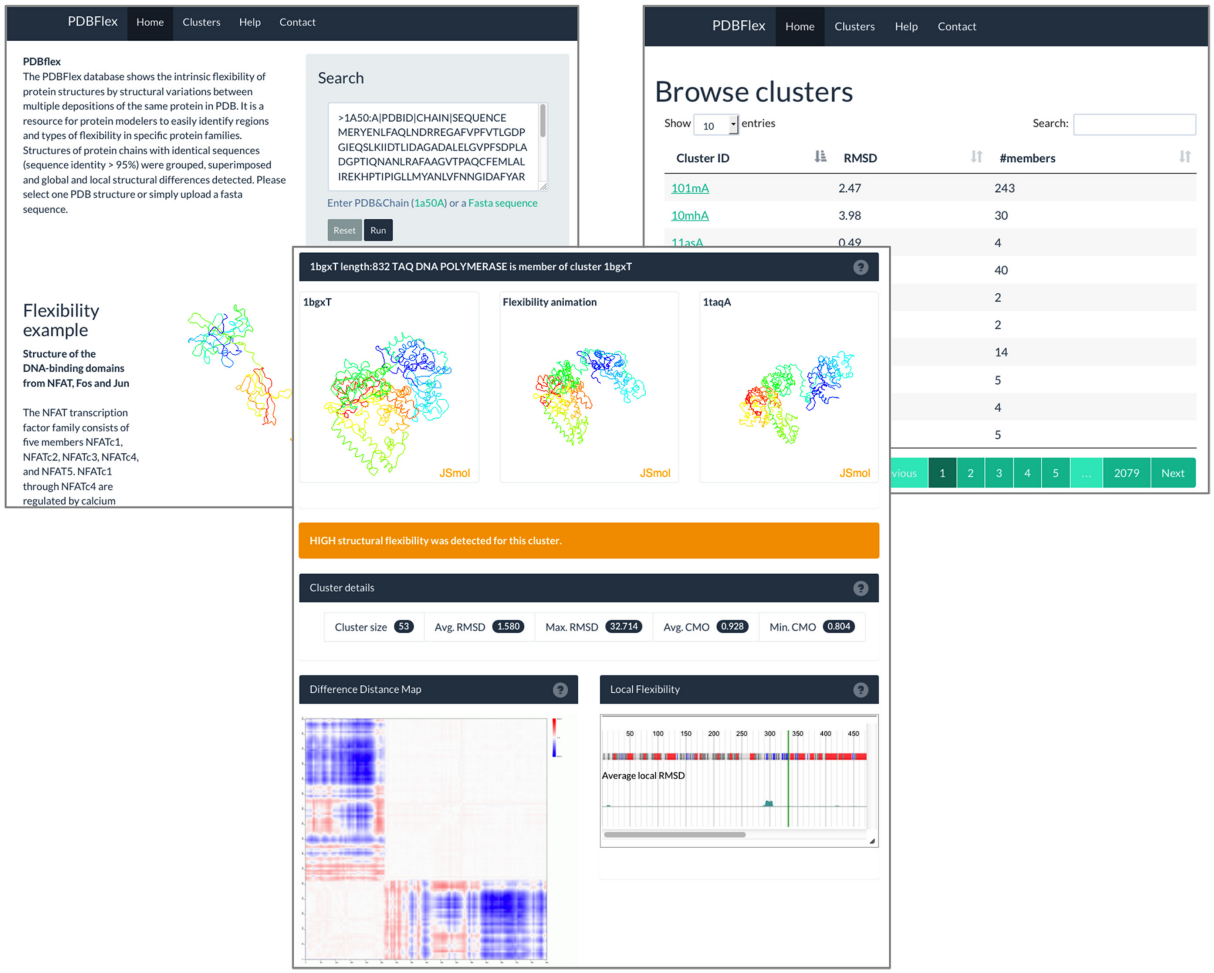
Cluster overview PDBFlex page provides information and several visualization options for each cluster of PDB chains with identical (within the threshold) sequences. The user can access this page either by providing the PDB id or a sequence of a protein to be analyzed (for instance 2hphA) or by browsing the list all available clusters (this list can be sorted either by Cα RMSD or by cluster size). The middle panel shows the cluster overview page for cluster of conformations representing the structure of the *E. coli*d-galactose-binding periplasmic protein, an example that would be used throughout the text. The top row shows the two most diverse structures in the cluster (left and right JSmol panels) and an animation of the transition between them (central JSmol panel). Displayed below are basic numerical data about the cluster. The Distance Difference Map illustrates the details of the structural rearrangement between the two most diverse conformations and the local flexibility preview presents local Cα RMSD variation along the sequences of the cluster members.

Coordinates representing all instances of independent depositions for a specific protein form a cluster that is analyzed for structural diversity. As discussed in the methods section, we use a threshold of 95% identity to define ‘identical’ proteins. This allows us to compare proteins with slightly different constructs boundaries or those that were solved with or without crystallization tags, the downside being that occasionally proteins from closely related species (e.g. human and mouse) can be included in one cluster. Data on the specific PDBFlex cluster can be accessed in two ways: (i) by specifying a PDB ID chain ID for a specific protein or (ii) by providing a sequence of a protein (Figure 1). For the first option, the server will simply display an overview the cluster containing this set of PDB coordinates, while for the second option the input sequence is aligned to all sequences of structures available on the server by a BLAST algorithm (13) and PDB IDs with matching sequences are displayed, sorted by sequence similarity. This option allows protein modelers to reason about the structural flexibility of a novel protein by analyzing such flexibility in several of its homologs.

### Browsing the cluster list

As another option, we provide direct access to the list of all clusters stored in the database. At this point only clusters with more than 3 members are analyzed and displayed. Here, users can browse through all clusters and select them either by size or by maximum Cα RMSD in a cluster.

### Cluster overview page

The cluster overview displays the properties of the query PDB chain and details of the cluster it belongs to. It automatically classifies the cluster based on the detected max. RMSD, based on the distribution we detected for all clusters (Figure 3). It shows the two most diverse structures in the cluster (i.e. a pair of structures with the highest Cα RMSD) and a morphing animation illustrating transition between these two structures. The Difference Distance Matrix (DDM) comparing these two structures is also shown as an additional visualization of structural variability.

**Figure 2.**
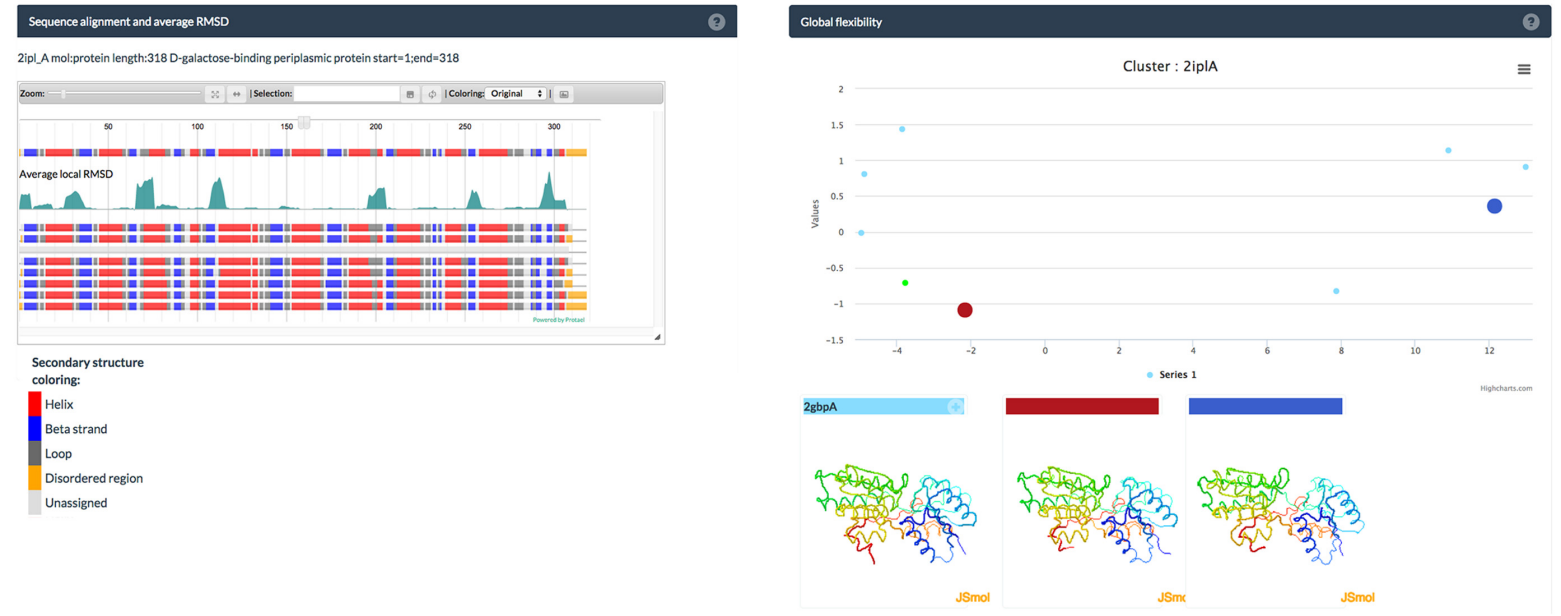
Detailed information about a structural diversity in a cluster representing a single protein. Left panel: Local flexibility view visualization based on master-slave, multiple-sequence-alignment of all cluster members. The top row in the view shows the secondary structure of the master sequence. Rows below correspond to aligned sequences of cluster members. The green curve below the master sequence shows average local Cα RMSD in the cluster (peaks in the curve correspond to regions of high local structural diversity in the cluster). Right panel: Global flexibility view based on Cα RMSD values calculated from all-against-all alignment of cluster members. Large, colored circles in the plot indicate centers of sub-clusters with their respective structures shown below. The user can search for and select any PDB structure on the plot, highlight structures with specific ligands or select a few structures for further visual inspection.

**Figure 3.**
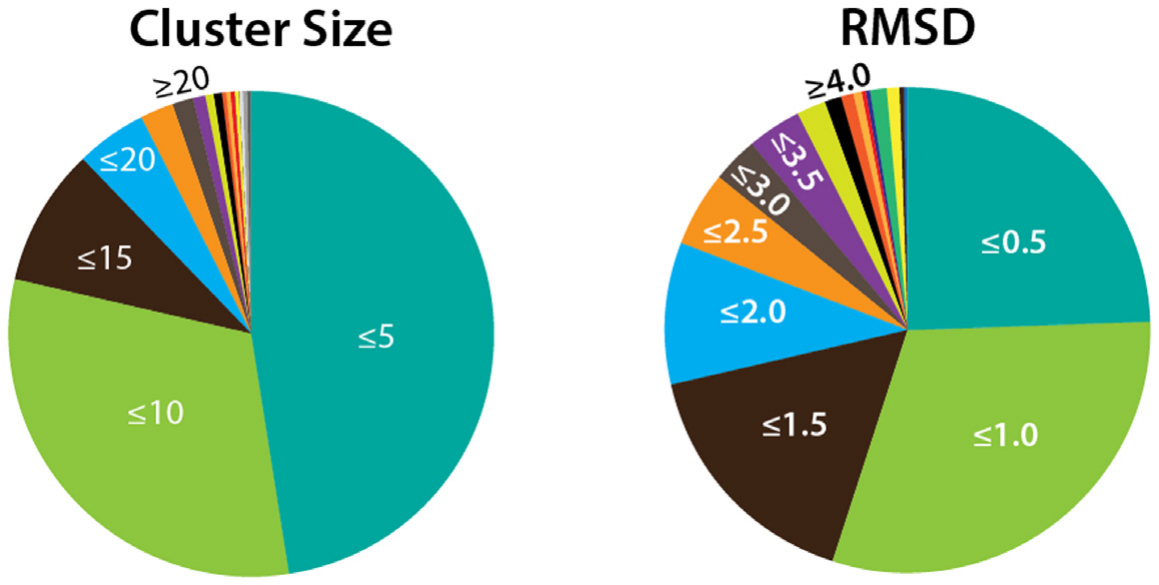
PDBFlex statistics. Approximately 47% of all clusters have 5 or less members and ∼78% of all clusters have 10 or less PDBs. 25% of out of all clusters show very low variability with a maximal Cα RSMD of less than 0.5Å, while ∼15% of all clusters have maximum Cα RSMD above 3Å.

The cluster overview also provides basic information such as cluster size, average and maximum Cα RMSD and average and minimum Contact Map Overlap (CMO) in the cluster. While the animation and DDM illustrate differences between the two most structurally diverse cluster members, a preview of local flexibility analysis provides information on the local structural diversity of cluster members presented as local RMSD (by clicking on this preview users can open Local Flexibility view to see more detailed information on local structural diversity, including secondary structure changes, shown in the left panel of Figure 2).

### Local flexibility view

The *Local Flexibility* view displays sequence and secondary structure variations within a cluster and is based on our new protein information viewer PROTAEL (14). The view is based on master-slave alignment of all sequences in the cluster, with the representative member (a ‘master’) placed on the top. The graph below shows average local Cα RMSD between the master and cluster members over the 10-residue window. The local structures of cluster members (secondary structures and structural disorder) are displayed below this graph. To speed up loading of the local flexibility page for large clusters, information for no more than 20 proteins, selected by the maximal diversity of the local structure, is shown by default. Full list can also be loaded.

Sequence coloring is based on the secondary structure and disorder information retrieved from PDB (see the data acquisition section). Helices are colored in red, beta strands in blue, loops in dark grey and orange represents disordered regions. Users can select a different coloring scheme using the drop-down menu in the view toolbar. The slider allows users to zoom into interesting regions (for instance parts with high local RMSD). Finally, the views can be exported as high-resolution figures.

### Global flexibility view

The *Global Flexibility* view visualizes global structural similarities and differences in a cluster. We used the compact, two-dimensional view to visualize structural flexibility in a cluster (instead of, for instance, hierarchical trees). All centers of structural sub-clusters are highlighted in the two-dimensional visualization graph and corresponding structures are shown in 3D in JSmol panels. Additionally, users can inspect any structure by clicking a point on the two-dimensional visualization graph - the corresponding structure is then displayed in the JSmol panel.

#### Selecting structures

Users have an option to select multiple structures for detailed analysis by clicking the ‘+’ symbol in the JSmol panel. All structures from the list of selected chains can be then displayed either in separate views or superposed in one view.

#### Ligand menu

Users have an option to highlight all cluster members that contain a specific ligand. We used the BioLiP database to identify all biologically relevant ligands associated with PDB chains (15). Cluster members with identical ligands can be highlighted in the two-dimensional visualization of structural sub-clusters to allow analysis of their distribution in the cluster.

#### Synchronized structure view

In the initial view, PDBFlex provides a visualization of all set of coordinates in the same relative position, as identified by structural superposition. The rotations of structures in individual windows can also be synchronized, similarly to an option implemented in on our POSA server (16). The user has the option to turn on synchronized rotation of all structure views to investigate large differences between compared structures in a cluster.

### Help pages

In order to help making the most of PDBFlex, we provide a series of help pages describing cluster size, local flexibility and global flexibility and other topics. These pages are available via the ‘Help’ menu from each page and most of the individual panels.

## DATA ACQUISITION

All sets of PDB coordinates determined by X-ray crystallography and containing more than 25 residues were included in the analysis (at this point structures solved by NMR spectroscopy are NOT included for technical reasons, they will be included in future versions of the PDBFlex database). Structures containing continuous fragments of more than 20 unidentified residues were excluded. We furthermore removed PDB-chains with no more than 25 coordinates of C***α*** atoms. This resulted in a list of 239,006 chains that were processed further.

### Clustering the sequences and finding representatives

We clustered the sequences of protein constructs deposited at PDB (using PDB SEQRES records to extract the sequences) at 95% sequence identity using cd-hit with the recommended ‘global sequence identity’ setting (17). As discussed earlier, this threshold was used to include proteins solved with slightly different constructs boundaries or those that were solved with or without crystallization tags in the same cluster, the downside being that occasionally proteins from closely related species (e.g. human and mouse) can be included in one cluster. The sequences of proteins from each cluster were aligned all-to-all with blastp (18). Alignments were then corrected to account for residues with unresolved coordinates in PDB entries and used to calculate Cα RSMD distances between all pairs of protein chains in each cluster. The blast alignments were also used to calculate a master-slave, multiple-sequence-alignment (msMSA) between the cluster representative (the ‘master’ sequence) and sequences of other cluster members (‘slave’ sequences). The longest (most complete) sequence in each cluster is used as a master. The msMSA alignment is then used for visualizations of local structure flexibility in the cluster.

### Data for the local flexibility viewer

We obtained information about secondary structure assignments from PDB (4), which uses a modified DSSP algorithm (19). Disordered (unresolved) structure regions from the PDB database for each protein chain were identified by lack of coordinates corresponding to the SEQRES defined sequences. For each cluster, we calculated local C*α* RSMD distances over 10-residue windows between the cluster representative and other cluster members and used them to estimate average local structure flexibility in the cluster. The average local structural flexibility is presented as a graph in the local flexibility viewer.

### Data for the global flexibility viewer

Cα RSMD values calculated for each pair of PDB chains in each cluster were used to prepare two-dimensional clustering visualization using the SciPy http://www.scipy.org/ (20) implementation of the Multi-Dimensional Scaling approach (21). The result was then further analyzed with the Mean Shift algorithm to automatically detect sub-clusters (22). By using this approach we were able to circumvent the otherwise tedious hierarchical clustering dependent on specific threshold selection for sub-cluster detection.

### Structure interpolation algorithm

The interpolation between similar structures was performed using our in-house algorithm based on Distance Matrices (DDMs) extrapolation (Rotkiewicz et. al, in preparation). In the first step of this approach intermediate distance matrices are calculated by linear interpolation between distance matrices of the two aligned structures (only aligned regions are taken into account). Subsequently, the intermediate structures are reproduced by energy gradient minimization with a reduced representation force field, while the approximate intermediate distance matrix is used as a set of harmonic constraints guiding the minimization. Thus, in contrast to a simple morphing, the structure changes are interpolated while preserving protein-like geometry of intermediate structures and internal structure of the structurally conserved rigid regions.

## EXAMPLES

In the figures we used the cluster of *E. coli* D-galactose binding periplasmic protein (23). This family is a particularly interesting example because bacterial periplasmic proteins are known to undergo dramatic, ligand-induced conformational changes upon substrate binding, initiating activation of the ABC transporters (23).

The ‘master’ structure representing this cluster is 2iplA and the cluster contains 9 structures. The family has an average Cα RMSD of 1.89Å and the most dissimilar pair (2qw1A, 2hphA) has a Cα RMSD of 4.29 Å.

The animation between the 2qw1A and 2hphA reveals the transition between open and closed conformation of the two domains.

The local flexibility view for cluster 2iplA reveals several local peaks, mainly representing loop-regions in the structure. The loop-regions around residues 70 and 110 in 2iplA, highlighted by these peaks, are in direct proximity to the ligand-binding region of the protein. Interestingly, two other loop regions with high RMSDs around residues 200 and 250 reside on regions opposite of the binding region.

In the global flexibility view structures in closed conformation cluster and form the larger sub-cluster of 6 structures (represented by 2iplA) while 2qw1A, 2fw0A and 2fvyA (represented by 2fw0A) form a small sub-cluster of ‘open’ confirmations (Figure 2).

## CONCLUSIONS AND FUTURE DEVELOPMENTS

We developed PDBFlex to present up-to-date information about structural flexibility in proteins revealed by differences between experimentally characterized structures of the same protein chain in the PDB database. Using our pipeline, we clustered all PDB chains at 95% sequence identity cutoff and then analyzed the resulting clusters independently. PDBFlex users can thoroughly examine structural flexibilities by selecting specific, interesting structures. Moreover, submitting sequences of unresolved structures to our server allows users interested in protein modeling to estimate structural flexibility for their protein sequence.

Our planned developments of the database will include (i) adding another clustering level at 40% sequence identity and creating links between high similarity (95%) clusters and low similarity clusters (40%) and (ii) adding an automated mechanism of weekly updates to process all new submissions from the PDB database.
